# Therapeutic Approaches to Limit Hemolysis-Driven Endothelial Dysfunction: Scavenging Free Heme to Preserve Vasculature Homeostasis

**DOI:** 10.1155/2013/396527

**Published:** 2013-05-27

**Authors:** Francesca Vinchi, Emanuela Tolosano

**Affiliations:** Department of Molecular Biotechnology and Health Sciences, Molecular Biotechnology Center, University of Torino, Via Nizza 52, 10126 Torino, Italy

## Abstract

Hemolysis results in the release of hemoglobin and heme into the bloodstream and is associated with the development of several pathologic conditions of different etiology, including hemoglobinopathies, hemolytic anemias, bacterial infections, malaria, and trauma. In addition, hemolysis is associated with surgical procedures, hemodialysis, blood transfusion, and other conditions in which mechanical forces can lead to red blood cell rupture. Free plasma hemoglobin and heme are toxic for the vascular endothelium since heme iron promotes oxidative stress that causes endothelial activation responsible for vasoocclusive events and thrombus formation. Moreover, free hemoglobin scavenges nitric oxide, reducing its bioavailability, and heme favours ROS production, thus causing oxidative nitric oxide consumption. This results in the dysregulation of the endothelium vasodilator:vasoconstrictor balance, leading to severe vasoconstriction and hypertension. Thus, endothelial dysfunction and impairment of cardiovascular function represent a common feature of pathologic conditions associated with hemolysis. In this review, we discuss how hemoglobin/heme released following hemolysis may affect vascular function and summarise the therapeutic approaches available to limit hemolysis-driven endothelial dysfunction. Particular emphasis is put on recent data showing the beneficial effects obtained through the use of the plasma heme scavenger hemopexin in counteracting heme-mediated endothelial damage in mouse models of hemolytic diseases.

## 1. Hemolytic Diseases

Hemolysis is a pathologic condition characterized by the increased release of hemoglobin (Hb) and heme. Several human diseases and pathologic situations with different etiology are associated with hemolysis including paroxysmal nocturnal hemoglobinuria (PNH), sickle-cell disease (SCD), thalassemias, hereditary spherocytosis and stomatocytosis, microangiopathic hemolytic anemias, pyruvate kinase deficiency, ABO mismatch transfusion reaction, paroxysmal cold hemoglobinuria, severe idiopathic autoimmune hemolytic anemia, infection-induced anemia, and malaria [1, 2]. Moreover, several recent studies indicate that hemolysis is also associated with procedures including hemodialysis, blood transfusion, and cardiac bypass in which mechanical shearing forces may lead to red blood cell rupture [3]. 

During hemolysis, red blood cells release Hb, which form stable complexes with the acute phase protein haptoglobin (Hp) [4]. The Hp-Hb complexes are cleared from circulation by monocytes and macrophages expressing the scavenger CD163 receptor. The function carried out by Hp is crucial, as demonstrated by studies on animal models and humans (recently reviewed in Schaer et al. [5]). When Hp's buffering capacity is overwhelmed, Hb undergoes a rapid conversion to metHb, liberating heme. Ferriheme then binds to albumin and other plasma components including lipoproteins and is subsequently transferred to hemopexin (Hx) [6, 7]. Hp and Hx, by binding with high affinity Hb and heme, respectively, block their prooxidant effects [4, 8]. Heme that escapes the binding to Hx enters into cells and is neutralized by heme oxygenases (HO). HO degrades the heme ring into iron, carbon monoxide (CO), and biliverdin, thus exerting primary anti-inflammatory, antioxidant, and antiapoptotic effects [2, 9–11]. In mammals, biliverdin is then rapidly converted into bilirubin by biliverdin reductase and excreted into the bile [12]. To date, three isoforms of HO have been identified, HO-1, HO-2, and HO-3, encoded by three different genes. The expression, distribution, and regulation, of HO-1, HO-2 and HO-3 differ among cell types and tissues. HO-3 has poor heme degrading capacity [13] and is now considered a pseudogene, whereas HO-1 and HO-2 are the actual heme-degrading enzymes [14]. HO-1 levels have been demonstrated to be low under normal physiological conditions but highly inducible by several stimuli including heme and other oxidant agents, while HO-2 has been described as a constitutively expressed enzyme [2, 15, 16]. The activity of HO is strictly associated with the function of ferritins and cytosolic proteins that sequester iron coming from heme catabolism. Ferritins are composed of varying ratios of two different subunits: H-ferritin and L-ferritin. H-ferritin is endowed with a ferroxidase activity and is essential for iron incorporation into the core of large L-ferritin and H-ferritin complexes [17]. 

In hemolytic diseases, cell-free plasma Hb and heme overwhelm homeostatic systems in place to remove them. As a consequence, various hemolytic diseases of different etiology share hemoglobinemia-related sequelae, characterized by endothelial dysfunction, thrombosis, vascular disease, and renal failure [14]. Observations from the clinical administration of artificial, purified, and recombinant Hb solutions have provided support for the causal relationship between excess cell-free Hb/heme in the bloodstream, symptoms, and cardiovascular events. In particular, pulmonary hypertension (PH) is emerging as one of the leading causes of morbidity and mortality in patients with hemolytic anemias, including SCD, thalassemia, PNH, hereditary spherocytosis and stomatocytosis, microangiopathic hemolytic anemias, pyruvate kinase deficiency, and possibly malaria [18–26].

In the last decades, medical advances in the management of patients suffering from SCD, thalassemia and other hemolytic anemias have led to significant increase in life expectancy [27]. Improved public health with neonatal genetic screening, parental and patient education, advances in red cell transfusion medicine safety, aggressive iron chelation therapy, penicillin prophylaxis for children under 6 years of age, immunization, and hydroxyurea therapy has all likely contributed to this effect on longevity [28]. Now, as a generation of patients with SCD and thalassemia ages, new chronic vascular complications of these hemoglobinopathies develop.

Here, we first discuss the causal relationship between hemolysis and endothelial dysfunction, and then we focus on different therapeutic approaches aimed at counteracting hemolysis-driven adverse effects on the vasculature.

## 2. Hemolysis and Endothelial Dysfunction

Chronic intravascular hemolysis is associated with prooxidant and proinflammatory stresses and with a state of endothelial dysfunction characterized by reduced nitric oxide (NO) bioavailability [29–31] and coagulopathy [32], leading to vasomotor instability and ultimately to vasculopathy [33]. The causal link between endothelial dysfunction and hemolysis has been well documented. Endothelial dysfunction is the earliest clinically detectable stage of cardiovascular disease characterized by a shift of the actions of the endothelium toward reduced vasodilation, a proinflammatory state, and prothrombotic properties [29, 34–37]. 

The entire circulatory tree is lined by a single epithelial-like layer of vascular endothelial cells (ECs). Although ECs exhibit characteristics that vary with anatomic location, both by organ system and by vessel type (e.g., artery, arteriole, capillary, venule, and vein), all ECs share common features that distinguish them from other cell types and allow them to perform critical homeostatic functions. Key homeostatic functions include retaining blood fluid, regulating blood flow, regulating macromolecule and fluid exchange with the tissues, preventing leukocyte activation, and aiding in immune surveillance for pathogens [38]. Injury or cell death impairs or prevents conduct of these activities, resulting in dysfunction. Most endothelial cell death is apoptotic, involving activation of caspases [39, 40]. 

Stimuli that can cause endothelial injury or death include oxidative stress, endoplasmic reticulum stress, metabolic stress, and genotoxic stress, as well as pathways of injury mediated by the innate and adaptive immune systems [41]. Hemolysis is directly involved in endothelial injury, and recent data suggest that chronic intravascular hemolysis is associated with a state of endothelial dysfunction, leading to hypertension [29, 30, 42, 43]. Some of these vascular effects, predisposing to hemolysis-associated hypertension, are linked to cytotoxic, proinflammatory, and prooxidant effects of iron-containing Hb and heme, whereas others are related to NO scavenging by excess plasma Hb [18, 44]. 

### 2.1. Hemolysis-Driven Oxidative Stress and Endothelial Activation

Because of chronic hemolysis, hemolytic patients are exposed to reactive oxygen species (ROS) generation catalyzed by heme-derived redox-active iron, the vessel wall being the primary exposed tissue. In the last years, it has been demonstrated that oxidative stress and inflammation directly contribute to vasoocclusive events and thrombus formation in hemolytic patients. Belcher and coauthors proposed that Hb, heme, and iron derived from hemolytic red blood cells promote excessive ROS production, leading to endothelial activation and adhesion molecule expression on the vessel wall, which in turn favour the adhesion of red blood cells and leukocytes to the endothelium, thus resulting in vascular instability and eventually vasoocclusion [42, 43]. Intravital microscopy studies on SCD mice demonstrated the critical role of adhesion molecules, such as P-selectin, VCAM-1, and ICAM-1, for the interaction of sickle red blood cells and leukocytes with the vessel wall and that blockade or knockout of these molecules prevents either cytokine- or hypoxia-induced stasis. In *β*-thalassemia, studies have reported increased levels of vascular adhesion molecules, indicating that endothelial dysfunction participates in thrombotic events.

### 2.2. Hemolysis-Mediated NO Bioavailability Reduction

In hemolytic patients, hemolysis-driven endothelial dysfunction is characterized by reduced NO bioavailability and NO resistance. This leads to dysregulation of the endothelium-derived vasodilator:vasoconstrictor system, thus causing severe vasoconstriction [19, 33, 45, 46]. 

NO is a potent endogenous vasodilator produced in endothelium by the endothelial NO synthase (NOS) enzyme, thanks to the oxygen-dependent conversion of L-arginine to citrulline. Once produced, NO can diffuse from the endothelium to adjacent smooth muscle where it binds avidly to the heme moiety of soluble guanylate cyclase. This activates the enzyme, which in turn converts GTP to cGMP, activating cGMP-dependent protein kinases and producing vasodilation. In addition to this vasodilation, which is tonic in nature and controls approximately 25% of our resting blood flow, NO promotes general vascular homeostasis and health [47]. NO tonically downregulates transcription of endothelial adhesion molecule genes, such as VCAM-1, ICAM-1, P-selectin, and E-selectin [48]. Nitric oxide also inhibits platelet activation, tissue factor expression, and thrombin generation [49]. NO modulates the expression of endothelin receptors (promoting a vasodilator effect by increase in endothelial-endothelin receptor B expression) and decreases expression of endothelin 1, a potent mitogen and vasoconstrictor [50].

It is now well accepted that during hemolysis, cell-free oxyHb in the plasma functions as an NO scavenger, producing metHb and nitrate (NO^3−^) in a reaction that is fast and irreversible [5]. Normally, in the absence of hemolysis, the amount of NO scavenged by Hb is greatly limited by the Hb sequestration within the red blood cell plasma membrane. This sequestration produces major bulk diffusional barriers to NO, which include an unstirred layer around the red blood cell, an intrinsic membrane permeability barrier to NO, and a cell-free zone along the endothelium in laminar flowing blood. These combined barriers reduce the reaction rate of NO with intracellular Hb by approximately 1000-fold [51]. In hemolytic disorders, the diffusional barriers created by the red cell membrane that limits NO reactions with Hb are disrupted, and the cell-free plasma Hb destroys NO at a rate of 1000-fold faster than intraerythrocytic Hb, resulting in abnormally high rates of NO consumption, which produces a state of resistance to NO activity [37, 46, 51, 52]. Consequently, smooth muscle guanylyl cyclase is not activated, and vasodilation is impaired. In support of this mechanism, plasma from patients with SCD contains oxyHb, which reacts with and consumes micromolar quantities of NO and inhibits forearm blood flow responses to NO donor infusions [34]. Remarkably, kinetic models suggest that levels of plasma Hb as low as 1 *μ*M have the potential to impair endothelial NO signaling, and heme concentrations as low as 6 *μ*M have been found to impair NO-dependant vasodilation in vivo in SCD patients [34]. Similar effects of hemolysis on NO bioavailability and endothelial function have recently been reported in malaria [53].

In hemolytic disorders, consumption/inactivation of NO is accelerated due not only to Hb scavenging but even to chronic oxidative stress promoted by free heme. In these patients, heme-induced ROS production is implicated in NO consumption and formation of peroxynitrite and in NOS uncoupling [18, 54]. Finally, hemolysis also releases arginase-1 from red blood cells that react with NOS substrate, L-arginine, to form ornithine, effectively reducing L-arginine availability for NO production by endothelial NOS. This results in a perpetual activation of the vascular endothelium because of chronic oxidative stress, hemolysis, and reduced NO [55]. 

### 2.3. Hemolysis-Induced Thrombosis

In addition to the toxic effects above reported, excessive plasma Hb and heme may contribute to platelet activation and thrombosis. The infusion of cross-linked Hb increases platelet aggregation and adhesion in vivo on prothrombotic surfaces such as an injured vessel wall. Administration of heme in healthy volunteers is associated with thrombophlebitis, demonstrating that heme can cause vascular inflammation followed by vascular obstruction in vivo [56, 57]. Interestingly, the addition of cell-free Hb to human serum at concentrations of 0.2 to 2.0 g/dL causes a dose-dependent inhibition of the metalloprotease ADAMTS13, an enzyme critical in limiting platelet thrombus formation. The major untoward effects of plasma Hb and heme on platelet function are most likely mediated by the scavenging of NO. NO interacts with components of the coagulation cascade to downregulate clot formation. In particular, NO has been shown to inhibit platelet aggregation, induce disaggregation of aggregated platelets, and inhibit platelet adhesion through increasing cGMP levels [58]. In fact, NO donor drugs (S-nitrothiols) that increase systemic levels of NO have been shown to inhibit platelet aggregation [59]. Conversely, NO scavenging by Hb or the reduction of NO generation by the inhibition of arginine metabolism results in an increase in platelet aggregation [34]. In animal models, reduction of NO causes increases in fibrin split products and thrombin-antithrombin complexes leading to significant fibrin deposition and thrombus formation. Moreover, in a patient with L-arginine deficiency, reduced NO production is associated with increased thrombin-antithrombin complexes and fibrin split products, while reversal of NO deficiency with L-arginine causes a reduction in intravascular coagulopathy [33, 35, 60, 61].

**Figure 1:**
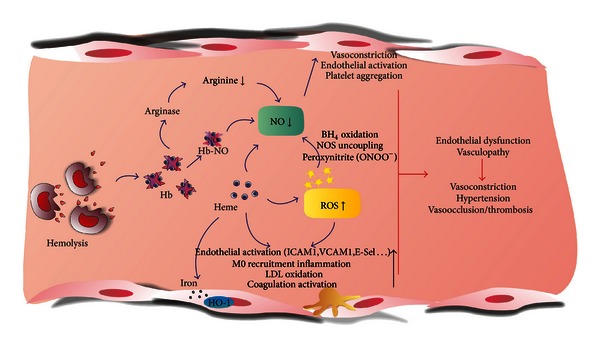
Hemolysis-driven endothelial toxicity. Free Hb/heme is responsible for reduced NO availability and ROS generation that contribute to endothelial dysfunction leading to vasoconstriction, hypertension, and vasoocclusion. See text for details.

The adverse effects of hemolysis on the vasculature are summarized in Figure 1. 

In the next two sections, we report the “state of the art” on the molecules used to limit Hb/heme toxicity on endothelium and then discuss our recent data on the therapeutic use of the plasma heme scavenger Hx.

## 3. Use of Therapeutic Molecules to Counteract Hb/Heme-Driven Endothelial Dysfunction

As stated above, the critical roles of endothelial activation, inflammation, and oxidative stress in the pathophysiology of hemolytic diseases have been well established. These studies open the possibility that the administration of antioxidants, inhibitors of endothelial activation, or agents able to restore NO homeostasis, could positively affect cardiovascular function in hemolytic diseases. Moreover, promising therapies are based on systems aimed at enhancing heme degradation and at scavenging Hb/heme from circulation. Several clinical trials testing the effect of such molecules are ongoing.

### 3.1. Antioxidants

The antioxidant niacin was found to inhibit vascular inflammation by decreasing endothelial ROS production and subsequent LDL oxidation and inflammatory cytokine production, key events involved in atherogenesis, highlighting vascular anti-inflammatory and potentially antiatherosclerotic properties for this molecule. Treatment with niacin was observed to improve endothelial dysfunction in patients with coronary artery disease and low high-density lipoproteins cholesterol. Niacin is expected to similarly act in SCD, causing an improvement in blood flow, and clinical trials are currently evaluating its therapeutical potential [62]. It has recently been demonstrated that niacin induces HO-1 and that the inhibition of HO activity attenuates the ability of niacin to inhibit vascular inflammation [63]. These data suggest that the protective effect of niacin on vascular function could be mediated by HO-1 and its products. Nevertheless, it should be taken into account that niacin/nicotinic acid is mildly hemolytic at pharmacologic doses [64], and therefore, it could exacerbate hemolytic damage thus rendering its use not so effective for the treatment of hemolytic patients.

The effect of the supplementation of another molecule, glutamine, in hemolytic patients is under evaluation [65, 66]. Glutamine is expected to improve the erythrocyte glutamine/glutamate ratio, a biomarker of oxidative stress, hemolysis, and PH in SCD and thalassemia patients and increase arginine bioavailability and subsequently alter sickle red cell endothelial interaction and clinical outcome [65, 67]. Additionally, oral glutamine is supposed to decrease biomarkers of hemolysis and adhesion molecules and improve the imbalanced arginine-to-ornithine ratio that occurs in hemolytic anemias, leading to improved arginine bioavailability and clinical endpoints of endothelial dysfunction and PH in patients with SCD and thalassemia [65, 66].

The protective effect of erythritol against endothelial dysfunction is also currently being tested. Erythritol, by inducing the expression of SOD2, activates the cell's own antioxidant machinery [68]. This activation or upregulation provides a protective effect to the endothelium and prevents endothelial dysfunction and hemolysis in the streptozotocin diabetic rat [68]. Thus, erythritol could be utilized to treat or prevent hypertension [68].

### 3.2. Adhesion Molecules Inhibitors

Recent studies suggest a beneficial effect for adhesion molecule inhibitors against hemolysis-induced vasculopathy. Among these molecules, the HDAC inhibitor trichostatin A and its analog suberoylanilide hydroxamic acid were found to markedly reduce endothelial activation and tissue factor expression in transgenic sickle mice, thus preventing vascular stasis [69]. 

Similarly, the small-molecule cyclic *α*V*β*3 and an anti-P-selectin aptamer decrease the adhesion of sickle red blood cells and leukocytes to endothelial cells in mouse model of SCD, suggesting a potential use of these molecules as novel therapeutic agents for vasculopathy associated with hemolytic pathologies [70, 71].

### 3.3. Agents Aimed at Restoring NO Homeostasis

In hemolytic patients, hemolysis-driven endothelial dysfunction is characterized by reduced NO bioavailability and NO resistance, leading to severe vasoconstriction. The effects of cell-free plasma Hb on NO consumption have been shown in clinical trials of Hb-based blood substitutes, in which various cell-free Hb preparations given to humans and animals showed dose-dependent effects on vasoconstriction, PH, and systemic hypertension. Induced intravascular hemolysis in a dog model promoted NO consumption and subsequent vasoconstriction and renal dysfunction [72]. This phenomenon has also been noted in patients with SCD. In these studies, the vasoconstrictive effects of NO depletion caused by hemolysis could be counteracted by inhaled NO, which reacts directly with the cell-free Hb in the pulmonary circulation, oxidizing it to metHb, which cannot then scavenge the NO systemically [73]. 

Additional studies provide evidence for a beneficial effect of inhaled NO and S-nitrosoalbumin on pulmonary injury induced by hypoxia/reoxygenation in a mouse model of SCD [74]. These observations provide new insights into the possible use of NO donors in the treatment of acute lung injury and vasoocclusive crisis in SCD. 

A role for arginine supplementation as a novel NO-based therapy for SCD has been proposed [75]. Arginine therapy increases NO bioavailability, thus resulting in improved microvascular function and reduced oxidative stress [75]. Consequently, arginine administration decreases pulmonary pressures in patients with SCD and secondary PH and inhibits endothelin-1-mediated activation of the Gardos channel in transgenic SCD mice, thus limiting erythrocyte dehydration and hemolysis [54, 76, 77].

Another development in our understanding of Hb-NO biology is the appreciation that Hb possesses a nitrite reductase and anhydrase activity that can convert nitrite to NO and N_2_O_3_, respectively, offsetting the Hb-dependent NO scavenging by vasodilation. Nitrite reacts with deoxygenated Hb to form metHb and NO. Consistent with this theory, recent studies have examined the addition of nitrite to Hb-based oxygen carriers and found that low concentrations of nitrite reverse the vasoconstrictive effects. According to this hypothesis, low doses of nitrite could be given before hemodialysis, cardiopulmonary bypass, or transfusion of aged blood to limit the cardiovascular toxicity of NO scavenging [78]. 

### 3.4. Enhanced Heme Degradation

In hemolytic patients, heme that accumulated in endothelial cells and in tissues is degraded by HO, mainly by the inducible HO-1 enzyme. HO-1 by exerting anti-inflammatory, antiproliferative, antiapoptotic, and antioxidant effects on the vasculature has been shown to protect against atherosclerosis [79]. Moreover, it promotes vascular repair after injury and prevents chronic allograft deterioration in heart transplantation [80, 81]. HO-1 protective effects are mediated by the products of heme catabolism, CO that is a strong vasodilator, and biliverdin/bilirubin that has antioxidant properties and can regulate the expression of protective genes [2]. In particular, biliverdin/bilirubin themselves scavenges multiple oxidants including superoxide, which would decrease NO bioavailability, induce peroxynitrite formation and contribute to dysregulation of endothelial function [82, 83]. SCD patients and mice showed an adaptive upregulation of HO-1 in response to hemolysis, and several data demonstrated the cardiovascular protective function of HO-1 [43, 84, 85]. Belcher and coauthors demonstrated that treatment of sickle mice with hemin to increase HO-1 expression inhibits hypoxia/reoxygenation-induced stasis, leukocyte-endothelium interactions, and NF-kappaB, VCAM-1, and ICAM-1 expressions. On the other hand, HO inhibition exacerbates stasis in sickle mice. Furthermore, treatment of sickle mice with the HO enzymatic product CO or biliverdin inhibits stasis and NF-kappaB, VCAM-1, and ICAM-1 expressions [43]. Another promising approach is based on the use of viral vectors or transposases to increase HO-1 expression [84, 86].

### 3.5. Plasma Hb Scavenging

Several studies reported the beneficial effects of Hp on inhibiting Hb toxicity [5, 72, 87]. A recent study published by Boretti et al. reports that cell-free plasma Hb has potent oxidative properties that are associated with hypertensive effects. They also found that increasing the levels of Hp in the circulation reduced this oxidative stress and limited both the hypertensive effects and the renal insufficiency associated with plasma hemoglobinemia [72].

Other studies reported the therapeutic use of Hp in animal models of transfusion-associated hemolysis [88]. Finally, Hp has already been used in several clinical trials in Japan.

The therapeutic use of Hp has been extensively reviewed by [5].

**Figure 2:**
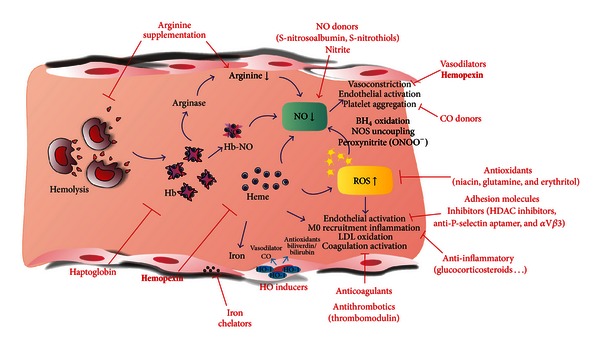
Therapeutic approaches aimed at counteracting Hb/heme toxicity. It is possible to use agents to restore NO availability, to limit ROS production and inflammation, to induce the protective HO-1 gene, to use Hb/heme scavengers to block Hb/heme adverse effects, or to chelate heme-derived prooxidant iron. In principle, combined therapies, by acting at different levels, could be even more effective.

The different therapeutic approaches discussed in this section are illustrated in Figure 2.

## 4. The Plasma Heme Scavenger Hemopexin

Hx is a glycoprotein mainly produced by the liver and released into plasma (plasma concentration: 0.5–1 mg/mL in humans and rodents) where it binds heme with very high affinity. Other than the liver, other sites of Hx synthesis are kidney mesangial cells, neurons, and glial cells of the central nervous system, Schwann cells, and ganglionic and photoreceptor cells of the retina and skeletal muscle [4, 89, 90]. Hx structure is characterized by two domains, resembling two thick disks that lock together at a 90° angle, joined by a linker peptide. The structure of Hx is characterized by its conserved clustering of histidine residues present in His-Gly sequences that coordinate heme [91]. The heme ligand is bound between the two domains of Hx in a pocket formed by the interdomain linker peptide. Heme binding and release result from opening and closing of the heme-binding pocket, through movement of the two domains and/or interdomain linker peptide [92]. 

Plasma level of Hx rises during the acute phase response following inflammation and heme overload [93]. Hx synthesis is mainly controlled at the transcriptional level. The human Hx promoter contains a specific cis-acting element, called Hpx A site, which is responsible for interleukin-6-mediated induction of Hx expression [94–96]. On the other hand, it is unknown whether heme directly controls Hx expression [4].

Studies in Hx-null mice demonstrated a crucial role for this protein in the protection against hemolytic damage. Hx-null mice are highly sensitive to phenylhydrazine-induced intravascular hemolysis and to heme overload obtained through intravenous injection of heme [8, 97, 98]. The Hx protective effect is particularly evident on the vasculature, the liver, and the kidney [8]. After heme overload, vessels of Hx-null mice showed an increased induction of adhesion molecules and suffered from oxidative stress. Moreover, NO availability after heme overload was reduced in Hx-null mice, and this was likely due to NOS uncoupling subsequent to heme-promoted ROS generation. Vessel damage was particularly evident in the liver of heme-overloaded Hx knockout mice, where sinusoids appeared congested. Finally, proinflammatory cytokines expression was increased in Hx-null mice after heme overload indicating that Hx can very efficiently limit heme inflammatory effects [8, 99].

Recently, we demonstrated that the Hx protective effect on the endothelium was achieved through the prevention of heme entry into endothelial cells (and/or heme intercalation in endothelial cell membranes) and the promotion of heme detoxification by the liver [99]. Hx very efficiently promotes heme uptake by hepatocytes that detoxify it through HO and direct excretion in the bile. This in vivo study confirms the previously reported in vitro results showing that treatment of hepatoma cell lines with heme-Hx complexes resulted in the induction of HO-1 and ferritins and thus in an efficient heme catabolism. Moreover, we were able to demonstrate that heme enters into endothelial cells if bound to albumin, but not if bound to Hx, and this was associated with the protection against heme-induced oxidative stress and to the prevention of adhesion molecules induction [99]. Similar results were obtained on macrophages as Hx also prevents heme entry into this cell type and macrophage activation (Vinchi et al., unpublished data). Interestingly, it has been reported that apo-Hx blocks the induction of IL-6 and TNF*α* in LPS-treated macrophages [100, 101]. This suggests that Hx can have an anti-inflammatory function independent from its heme scavenger function, likely by acting on TLR4-activated pathways.

## 5. Therapeutical Use of Hx to Limit Heme-Driven Endothelial Dysfunction in Hemolytic Disorders

We recently showed the effects of an Hx-based therapy in mouse models of two hemolytic disorders, SCD and *β*-thalassemia. Both these diseases, despite having different etiology and clinical settings, have high rate of intravascular hemolysis as a common feature. Both of these disorders can cause fatigue, jaundice, and episodes of pain ranging from mild to severe. Dysfunction of the liver, lung, kidney, and heart is common, and stroke may occur. Moreover, in severe forms of SCD and *β*-thalassemia, a transfusion regimen is necessary, and this further exacerbates Hb/heme toxicity [33].

Our data show that Hx infusion alleviates heme-induced endothelial activation, inflammation, and oxidative injury in mouse models, thus suggesting important implication for a therapeutic use of Hx in the treatment of hemolytic diseases (Figure 2). 

Hx treatment resulted in a marked improvement of the health status of anemic mice and preservation of their organ functionality. In particular, we demonstrated that Hx administration, by scavenging free heme, alleviates heme-induced ROS formation and tissue oxidative injury and limits the induction of adhesion molecules in SCD and *β*-thalassemia mice, thus indicating that Hx may confer protection against heme-driven oxidative stress as well as endothelial activation and inflammation [99]. Moreover, we showed that Hx therapy promotes NO production by reducing heme-induced oxidative consumption of NOS cofactors and NOS enzyme uncoupling. Increased bioavailability of NO suggests that Hx is able to positively affect vascular homeostasis, counteracting endothelial dysfunction associated with hemolytic pathologies [99].

Our recent data also indicate a strong antihypertensive role for Hx in hemolytic pathologies. Hx therapy strongly reduces blood pressure in hemolytic animals, counteracting heme-induced vasoconstriction. This is further supported by a marked decrease in cardiac output and aortic valve peak pressure observed in SCD mice following Hx therapy [99]. 

Additionally, Hx administration reduces proinflammatory cytokine production in hemolytic animals, supporting a strong anti-inflammatory role for this molecule [99]. Compared to other anti-inflammatory agents such as glucocorticosteroids that have a limited use in clinics because of their side effects and immunosuppressive impact, Hx, by acting as a physiologic heme chelator, is expected to be well tolerated. We did not observe deleterious effects of the Hx therapy on renal function in mice despite the fact that previous papers had described a protease activity of serum Hx responsible for proteinuria and glomerular alterations characteristic for minimal change nephrotic syndrome in the rat [102, 103]. This might be due to the different preparations of Hx used or, more likely, to the different pathologic models analysed. Our data support the conclusion that Hx is the most important heme scavenger under conditions of enhanced hemolysis as those experienced by SCD and thalassemic mice when plasma hemoglobin levels exceed haptoglobin binding capacity.

Hx exerts its protective effect mainly by enhancing HO expression and activity in the liver and by limiting HO-1 induction in vascular endothelium and likely in other extrahepatic tissues [99]. 

In SCD and thalassemic patients, excess iron deposition in the liver, further worsened by transfusion, may cause fibrosis/cirrhosis and hepatocellular carcinoma. Nevertheless, the beneficial effects due to Hx treatment observed in anemic mice support the idea that the organism can tolerate a greater iron burden in the liver that is a tissue predisposed to iron storage, while it is preferable to alleviate iron loading in tissues, such as the heart and kidney that are not usually predisposed to iron accumulation [104]. In addition, we demonstrated that Hx treatment also promotes heme excretion in the bile, thus providing a way for iron to directly leave the body [99]. This represents a mechanism on which to act in the future in order to enhance the liver heme detoxifying potential.

On the other hand, by redirecting heme to the liver, Hx limits heme uptake and prevents HO-1 induction as well as iron loading and activation of endothelial cells, thus reducing ROS production, inflammation, and, eventually, cell death. These results are in agreement with data showing that HO-1 transgene expression in the liver of sickle mice resulted in a decrease in markers of vascular inflammation and inhibition of vasoocclusion [84]. Indeed, an important outcome of Hx therapy is the prevention of iron accumulation in the vascular endothelium and in the heart. This has clinical relevance since hemolysis-driven iron overload, exacerbated by blood transfusions, strongly contributes to heart failure, one of the most common causes of death in thalassemia patients. Our results suggest that Hx administration could be beneficial in transfused patients to alleviate the myocardial iron burden. 

These data provide the rationale for the use of purified or recombinant Hx and/or for the design of Hx-based drugs, with high affinity for heme and rapidly cleared by the liver, which might be used pharmacologically as heme chelators to prevent heme-mediated tissue damage in patients suffering from hemolytic diseases.

## 6. Conclusions

Free Hb/heme resulting from hemolysis is responsible for reduced NO availability, oxidative stress, and inflammation, all contributing to vascular dysfunction. Therapeutic approaches are aimed at enhancing heme degradation, at counteracting heme prooxidant activity, at limiting endothelial activation, and at preserving NO homeostasis. Promising novel therapies are based on the use of plasma Hb and heme scavengers to prevent Hb/heme toxicity. In particular, the use of purified Hx in mouse models of SCD and *β*-thalassemia has provided the proof of principle of such approach. Future studies are needed to translate these results into clinical practice. Considering that the different approaches described in this review target different levels of the cascade of events starting with Hb/heme overload and ending with vascular dysfunction, it is time to speculate on the effectiveness of combined therapies. For instance, we expect that promoting Hb and heme scavenging with Hp and Hx, respectively, and simultaneously enhancing HO-1 expression may almost completely avoid heme accumulation and consequently its toxic effects. Similarly, the association of antioxidants and/or drugs aimed at restoring NO may improve the effectiveness of Hb/heme scavenging.
